# Supplementary material for: Relationship between the Young’s Moduli of Whole Microcapsules and Their Shell Material Established by Micromanipulation Measurements Based on Diametric Compression between Two Parallel Surfaces and Numerical Modelling
Source: Micromachines (Basel). 2023 Jan 1;14(1):123. doi: 10.3390/mi14010123 (PMC9867421; doi:10.3390/mi14010123)
Supplement: Supplementary file 1 [file micromachines-14-00123-s001.zip › micromachines-2096822-supplementary.pdf]

Supplementary Material

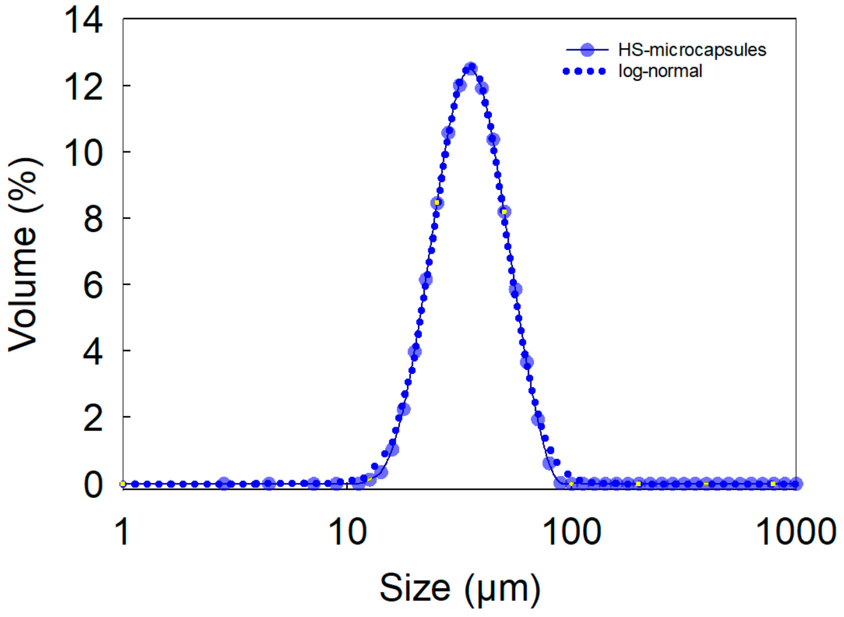

Figure S1. Particle Size Distribution of HS laden microcapsules (Master Sizer 2000, Malvern Instruments Ltd, Malvern, UK).

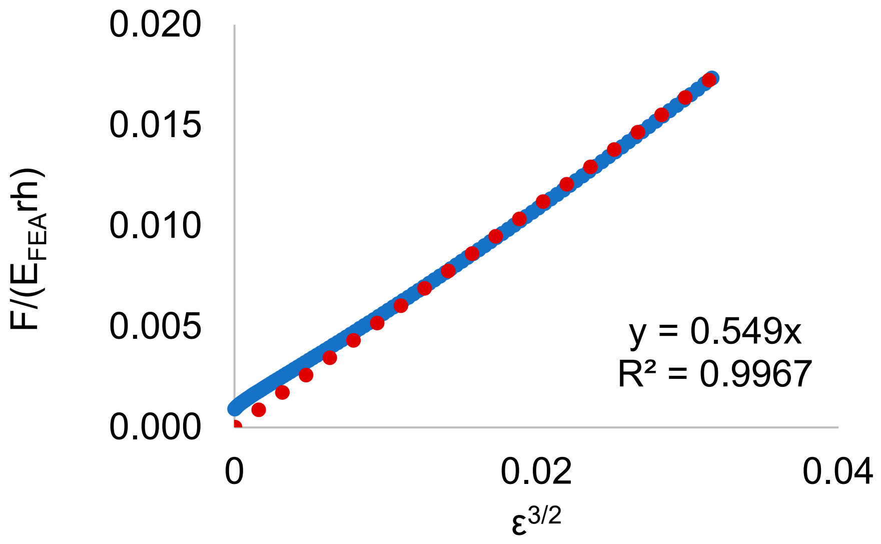

Figure S2. The Hertz model fitting results (pink line) of dimensionless force versus fractional deformation up to 10% deformation (h/r =0.05, from Figure 5B) where the slope is $f_4 = 0.549$.

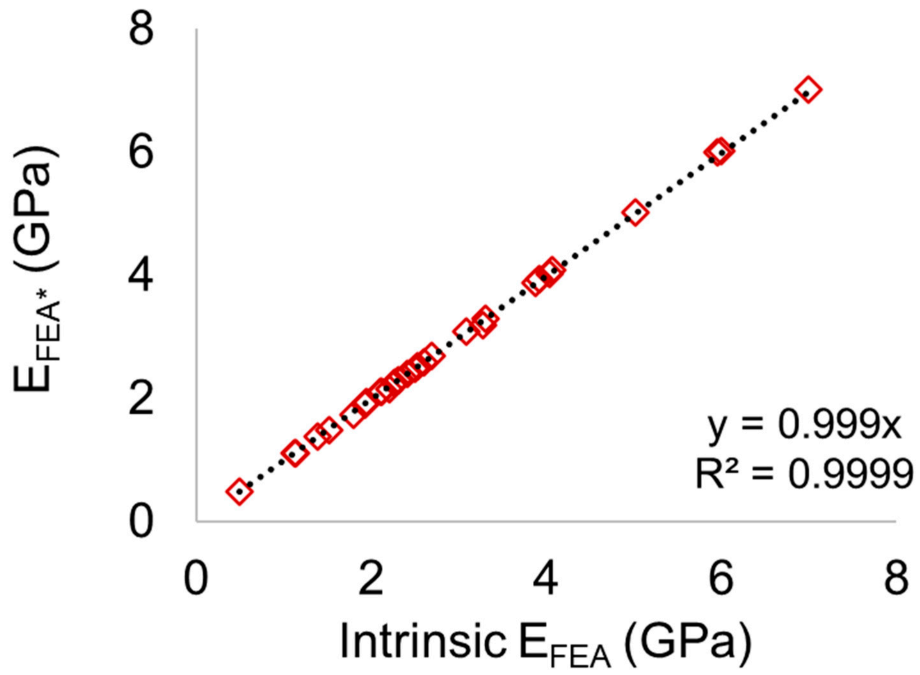

Figure S3. Model application example: synthetic microcapsules by Luo, et al. [1]; linear relationship between the FEA derived E_{FEA} and model φ-predicted Young's modulus E_{FEA}^*, with their average values of the shell material being 2.92 ± 0.29 GPa and 2.91 ± 0.29 GPa, respectively.

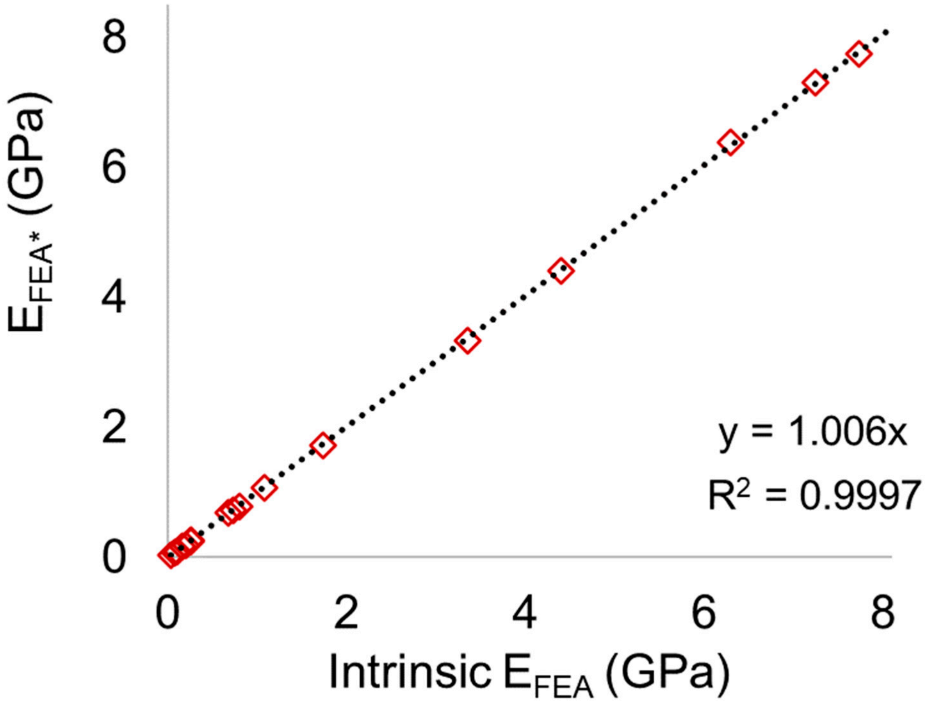

Figure S4. Model application example: double coated microcapsules by Baiocco, et al. [2]; linear relationship between the FEA derived E_{FEA} and model φ-predicted Young's modulus E_{FEA}^*, with their average values of the shell material being 2.59 ± 0.83 GPa and 2.61 ± 0.84 GPa, respectively.

References

1. Luo, S.; Gao, M.; Pan, X.; Wang, Y.; He, Y.; Zhu, L.; Si, T.; Sun, Y., Fragrance oil microcapsules with low content of formaldehyde: Preparation and characterization. *Colloids and Surfaces A: Physicochemical and Engineering Aspects* **2022,** 648, 129019.
2. Baiocco, D.; Preece, J. A.; Zhang, Z., Microcapsules with a Fungal Chitosan-gum Arabic-maltodextrin Shell to Encapsulate Health-Beneficial Peppermint Oil. *Food Hydrocolloids for Health* **2021**, 100016.
